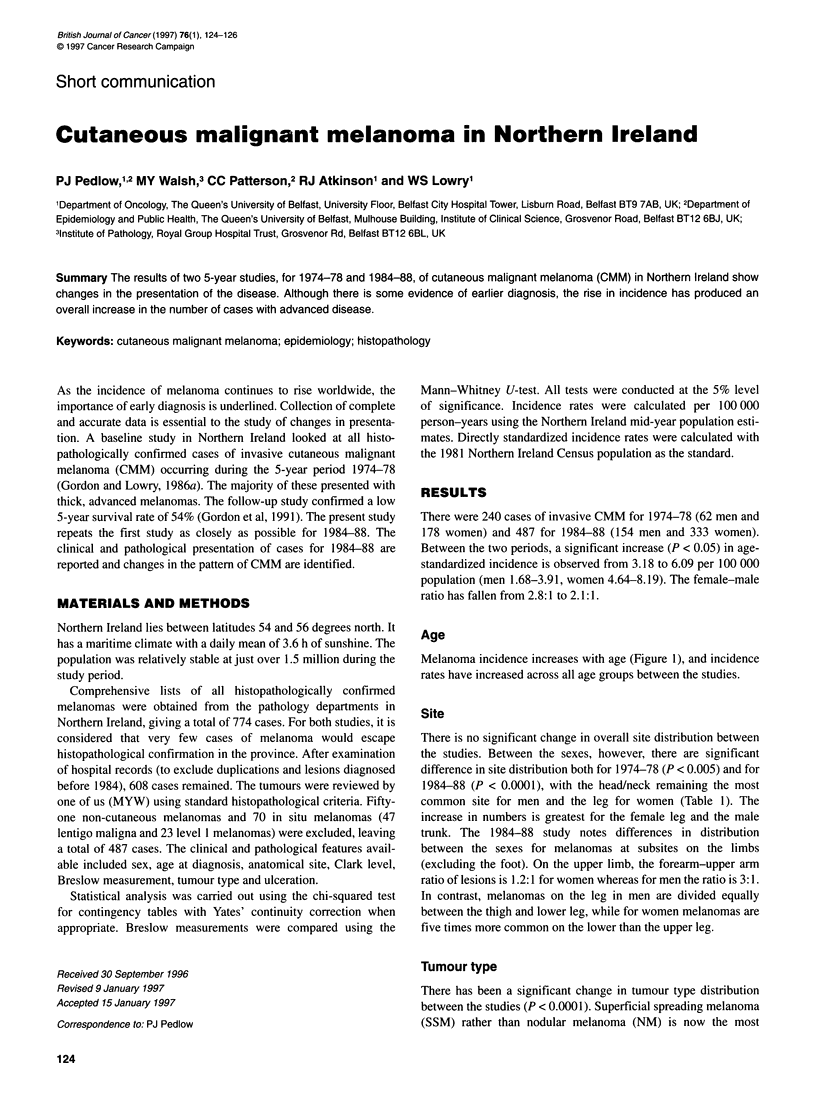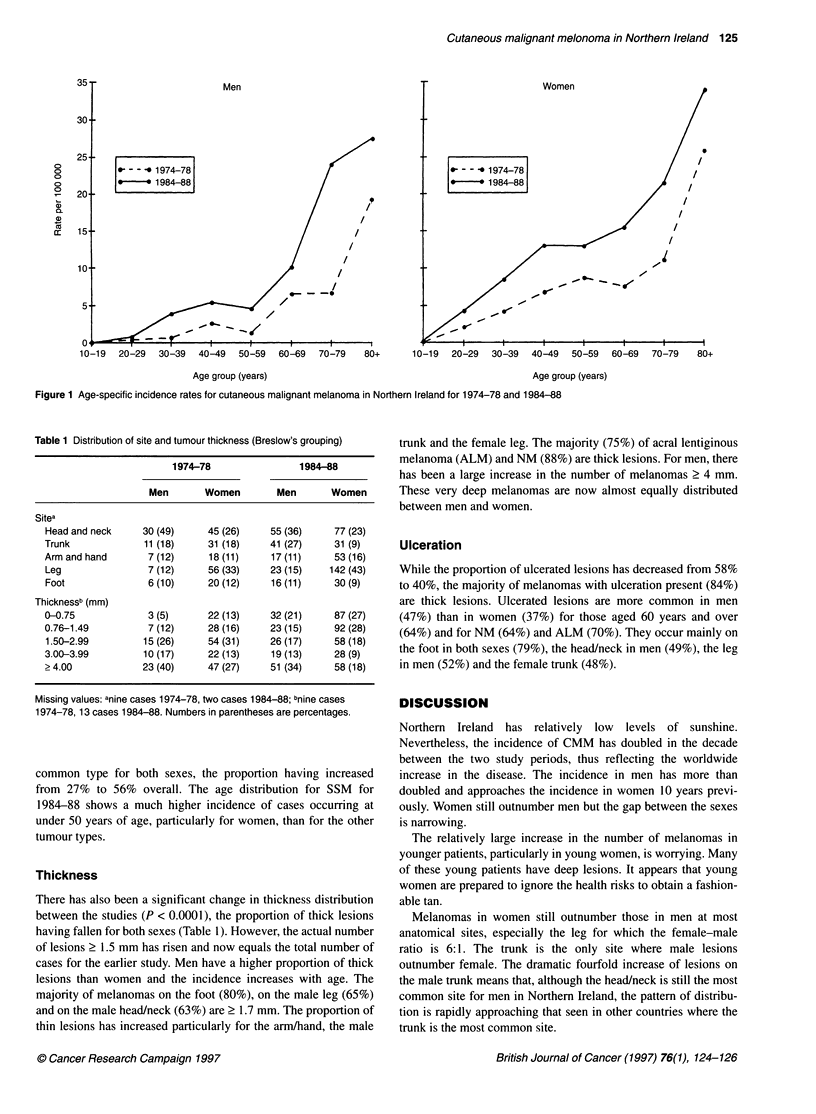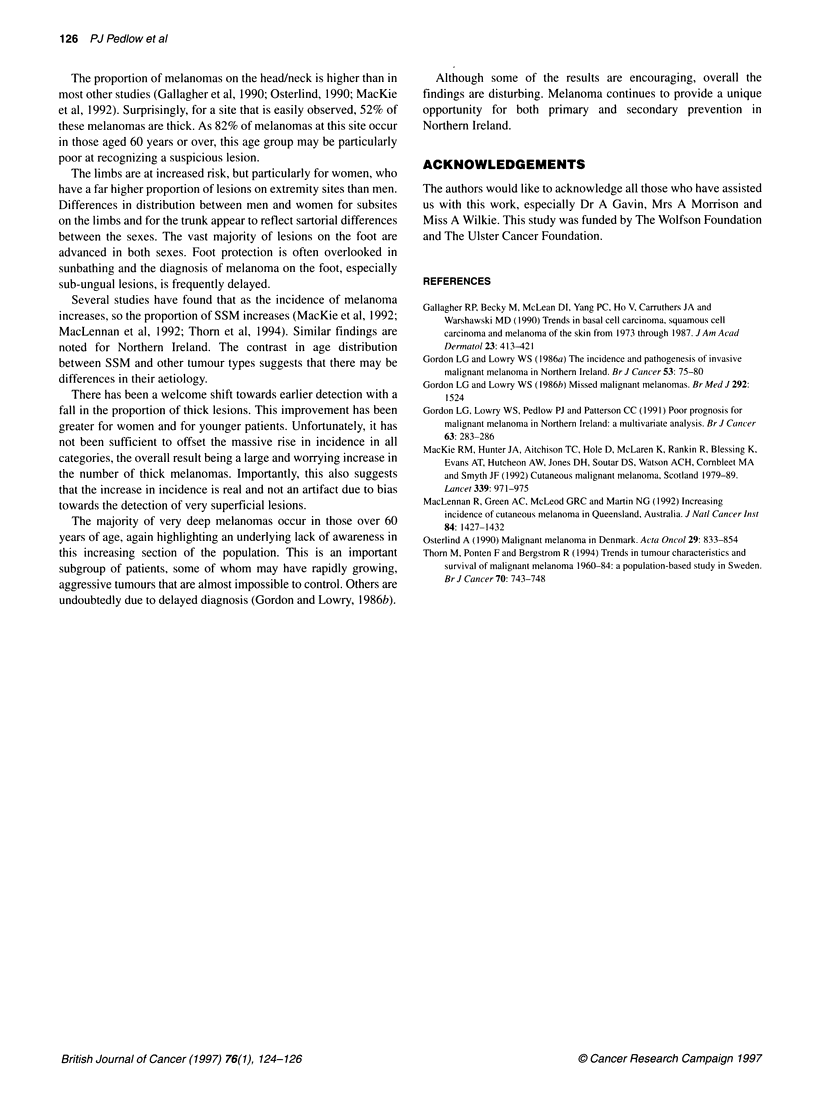# Cutaneous malignant melanoma in Northern Ireland.

**DOI:** 10.1038/bjc.1997.347

**Published:** 1997

**Authors:** P. J. Pedlow, M. Y. Walsh, C. C. Patterson, R. J. Atkinson, W. S. Lowry

**Affiliations:** Department of Oncology, The Queen's University of Belfast, Belfast City Hospital Tower, UK.

## Abstract

The results of two 5-year studies, for 1974-78 and 1984-88, of cutaneous malignant melanoma (CMM) in Northern Ireland show changes in the presentation of the disease. Although there is some evidence of earlier diagnosis, the rise in incidence has produced an overall increase in the number of cases with advanced disease.


British Journal of Cancer (1997) 76(1), 124–126
© 1997 Cancer Research Campaign

Short communication

### Cutaneous malignant melanoma in Northern Ireland

**PJ Pedlow,[1,2] MY Walsh,[3] CC Patterson,[2] RJ Atkinson[1] and WS Lowry[1]**

[1]Department of Oncology, The Queen's University of Belfast, University Floor, Belfast City Hospital Tower, Lisburn Road, Belfast BT9 7AB, UK; [2]Department of Epidemiology and Public Health, The Queen's University of Belfast, Mulhouse Building, Institute of Clinical Science, Grosvenor Road, Belfast BT12 6BJ, UK; [3]Institute of Pathology, Royal Group Hospital Trust, Grosvenor Rd, Belfast BT12 6BL, UK

**Summary** The results of two 5-year studies, for 1974–78 and 1984–88, of cutaneous malignant melanoma (CMM) in Northern Ireland show changes in the presentation of the disease. Although there is some evidence of earlier diagnosis, the rise in incidence has produced an overall increase in the number of cases with advanced disease.

**Keywords:** cutaneous malignant melanoma; epidemiology; histopathology

As the incidence of melanoma continues to rise worldwide, the importance of early diagnosis is underlined. Collection of complete and accurate data is essential to the study of changes in presentation. A baseline study in Northern Ireland looked at all histopathologically confirmed cases of invasive cutaneous malignant melanoma (CMM) occurring during the 5-year period 1974–78 (Gordon and Lowry, 1986*a*). The majority of these presented with thick, advanced melanomas. The follow-up study confirmed a low 5-year survival rate of 54% (Gordon et al, 1991). The present study repeats the first study as closely as possible for 1984–88. The clinical and pathological presentation of cases for 1984–88 are reported and changes in the pattern of CMM are identified.

### MATERIALS AND METHODS

Northern Ireland lies between latitudes 54 and 56 degrees north. It has a maritime climate with a daily mean of 3.6 h of sunshine. The population was relatively stable at just over 1.5 million during the study period.

Comprehensive lists of all histopathologically confirmed melanomas were obtained from the pathology departments in Northern Ireland, giving a total of 774 cases. For both studies, it is considered that very few cases of melanoma would escape histopathological confirmation in the province. After examination of hospital records (to exclude duplications and lesions diagnosed before 1984), 608 cases remained. The tumours were reviewed by one of us (MYW) using standard histopathological criteria. Fifty-one non-cutaneous melanomas and 70 in situ melanomas (47 lentigo maligna and 23 level 1 melanomas) were excluded, leaving a total of 487 cases. The clinical and pathological features available included sex, age at diagnosis, anatomical site, Clark level, Breslow measurement, tumour type and ulceration.

Statistical analysis was carried out using the chi-squared test for contingency tables with Yates' continuity correction when appropriate. Breslow measurements were compared using the Mann–Whitney *U*-test. All tests were conducted at the 5% level of significance. Incidence rates were calculated per 100 000 person–years using the Northern Ireland mid-year population estimates. Directly standardized incidence rates were calculated with the 1981 Northern Ireland Census population as the standard.

*Received 30 September 1996*
*Revised 9 January 1997*
*Accepted 15 January 1997*

*Correspondence to:* PJ Pedlow

### RESULTS

There were 240 cases of invasive CMM for 1974–78 (62 men and 178 women) and 487 for 1984–88 (154 men and 333 women). Between the two periods, a significant increase ($P < 0.05$) in age-standardized incidence is observed from 3.18 to 6.09 per 100 000 population (men 1.68–3.91, women 4.64–8.19). The female–male ratio has fallen from 2.8:1 to 2.1:1.

### Age

Melanoma incidence increases with age (Figure 1), and incidence rates have increased across all age groups between the studies.

### Site

There is no significant change in overall site distribution between the studies. Between the sexes, however, there are significant difference in site distribution both for 1974–78 ($P < 0.005$) and for 1984–88 ($P < 0.0001$), with the head/neck remaining the most common site for men and the leg for women (Table 1). The increase in numbers is greatest for the female leg and the male trunk. The 1984–88 study notes differences in distribution between the sexes for melanomas at subsites on the limbs (excluding the foot). On the upper limb, the forearm–upper arm ratio of lesions is 1.2:1 for women whereas for men the ratio is 3:1. In contrast, melanomas on the leg in men are divided equally between the thigh and lower leg, while for women melanomas are five times more common on the lower than the upper leg.

### Tumour type

There has been a significant change in tumour type distribution between the studies ($P < 0.0001$). Superficial spreading melanoma (SSM) rather than nodular melanoma (NM) is now the most

**Figure 1** Age-specific incidence rates for cutaneous malignant melanoma in Northern Ireland for 1974–78 and 1984–88

**Table 1** Distribution of site and tumour thickness (Breslow's grouping)

| | 1974–78 | | 1984–88 | |
|---|---|---|---|---|
| | Men | Women | Men | Women |
| Site[a] | | | | |
| Head and neck | 30 (49) | 45 (26) | 55 (36) | 77 (23) |
| Trunk | 11 (18) | 31 (18) | 41 (27) | 31 (9) |
| Arm and hand | 7 (12) | 18 (11) | 17 (11) | 53 (16) |
| Leg | 7 (12) | 56 (33) | 23 (15) | 142 (43) |
| Foot | 6 (10) | 20 (12) | 16 (11) | 30 (9) |
| Thickness[b] (mm) | | | | |
| 0–0.75 | 3 (5) | 22 (13) | 32 (21) | 87 (27) |
| 0.76–1.49 | 7 (12) | 28 (16) | 23 (15) | 92 (28) |
| 1.50–2.99 | 15 (26) | 54 (31) | 26 (17) | 58 (18) |
| 3.00–3.99 | 10 (17) | 22 (13) | 19 (13) | 28 (9) |
| ≥ 4.00 | 23 (40) | 47 (27) | 51 (34) | 58 (18) |

Missing values: [a]nine cases 1974–78, two cases 1984–88; [b]nine cases 1974–78, 13 cases 1984–88. Numbers in parentheses are percentages.

common type for both sexes, the proportion having increased from 27% to 56% overall. The age distribution for SSM for 1984–88 shows a much higher incidence of cases occurring at under 50 years of age, particularly for women, than for the other tumour types.

### Thickness

There has also been a significant change in thickness distribution between the studies ($P < 0.0001$), the proportion of thick lesions having fallen for both sexes (Table 1). However, the actual number of lesions ≥ 1.5 mm has risen and now equals the total number of cases for the earlier study. Men have a higher proportion of thick lesions than women and the incidence increases with age. The majority of melanomas on the foot (80%), on the male leg (65%) and on the male head/neck (63%) are ≥ 1.7 mm. The proportion of thin lesions has increased particularly for the arm/hand, the male trunk and the female leg. The majority (75%) of acral lentiginous melanoma (ALM) and NM (88%) are thick lesions. For men, there has been a large increase in the number of melanomas ≥ 4 mm. These very deep melanomas are now almost equally distributed between men and women.

### Ulceration

While the proportion of ulcerated lesions has decreased from 58% to 40%, the majority of melanomas with ulceration present (84%) are thick lesions. Ulcerated lesions are more common in men (47%) than in women (37%) for those aged 60 years and over (64%) and for NM (64%) and ALM (70%). They occur mainly on the foot in both sexes (79%), the head/neck in men (49%), the leg in men (52%) and the female trunk (48%).

### DISCUSSION

Northern Ireland has relatively low levels of sunshine. Nevertheless, the incidence of CMM has doubled in the decade between the two study periods, thus reflecting the worldwide increase in the disease. The incidence in men has more than doubled and approaches the incidence in women 10 years previously. Women still outnumber men but the gap between the sexes is narrowing.

The relatively large increase in the number of melanomas in younger patients, particularly in young women, is worrying. Many of these young patients have deep lesions. It appears that young women are prepared to ignore the health risks to obtain a fashionable tan.

Melanomas in women still outnumber those in men at most anatomical sites, especially the leg for which the female–male ratio is 6:1. The trunk is the only site where male lesions outnumber female. The dramatic fourfold increase of lesions on the male trunk means that, although the head/neck is still the most common site for men in Northern Ireland, the pattern of distribution is rapidly approaching that seen in other countries where the trunk is the most common site.

The proportion of melanomas on the head/neck is higher than in most other studies (Gallagher et al, 1990; Osterlind, 1990; MacKie et al, 1992). Surprisingly, for a site that is easily observed, 52% of these melanomas are thick. As 82% of melanomas at this site occur in those aged 60 years or over, this age group may be particularly poor at recognizing a suspicious lesion.

The limbs are at increased risk, but particularly for women, who have a far higher proportion of lesions on extremity sites than men. Differences in distribution between men and women for subsites on the limbs and for the trunk appear to reflect sartorial differences between the sexes. The vast majority of lesions on the foot are advanced in both sexes. Foot protection is often overlooked in sunbathing and the diagnosis of melanoma on the foot, especially sub-ungual lesions, is frequently delayed.

Several studies have found that as the incidence of melanoma increases, so the proportion of SSM increases (MacKie et al, 1992; MacLennan et al, 1992; Thorn et al, 1994). Similar findings are noted for Northern Ireland. The contrast in age distribution between SSM and other tumour types suggests that there may be differences in their aetiology.

There has been a welcome shift towards earlier detection with a fall in the proportion of thick lesions. This improvement has been greater for women and for younger patients. Unfortunately, it has not been sufficient to offset the massive rise in incidence in all categories, the overall result being a large and worrying increase in the number of thick melanomas. Importantly, this also suggests that the increase in incidence is real and not an artifact due to bias towards the detection of very superficial lesions.

The majority of very deep melanomas occur in those over 60 years of age, again highlighting an underlying lack of awareness in this increasing section of the population. This is an important subgroup of patients, some of whom may have rapidly growing, aggressive tumours that are almost impossible to control. Others are undoubtedly due to delayed diagnosis (Gordon and Lowry, 1986*b*).

Although some of the results are encouraging, overall the findings are disturbing. Melanoma continues to provide a unique opportunity for both primary and secondary prevention in Northern Ireland.

### ACKNOWLEDGEMENTS

The authors would like to acknowledge all those who have assisted us with this work, especially Dr A Gavin, Mrs A Morrison and Miss A Wilkie. This study was funded by The Wolfson Foundation and The Ulster Cancer Foundation.

### REFERENCES

Gallagher RP, Becky M, McLean DI, Yang PC, Ho V, Carruthers JA and Warshawski MD (1990) Trends in basal cell carcinoma, squamous cell carcinoma and melanoma of the skin from 1973 through 1987. *J Am Acad Dermatol* **23**: 413–421

Gordon LG and Lowry WS (1986*a*) The incidence and pathogenesis of invasive malignant melanoma in Northern Ireland. *Br J Cancer* **53**: 75–80

Gordon LG and Lowry WS (1986*b*) Missed malignant melanomas. *Br Med J* **292**: 1524

Gordon LG, Lowry WS, Pedlow PJ and Patterson CC (1991) Poor prognosis for malignant melanoma in Northern Ireland: a multivariate analysis. *Br J Cancer* **63**: 283–286

MacKie RM, Hunter JA, Aitchison TC, Hole D, McLaren K, Rankin R, Blessing K, Evans AT, Hutcheon AW, Jones DH, Soutar DS, Watson ACH, Cornbleet MA and Smyth JF (1992) Cutaneous malignant melanoma, Scotland 1979–89. *Lancet* **339**: 971–975

MacLennan R, Green AC, McLeod GRC and Martin NG (1992) Increasing incidence of cutaneous melanoma in Queensland, Australia. *J Natl Cancer Inst* **84**: 1427–1432

Osterlind A (1990) Malignant melanoma in Denmark. *Acta Oncol* **29**: 833–854

Thorn M, Ponten F and Bergstrom R (1994) Trends in tumour characteristics and survival of malignant melanoma 1960–84: a population-based study in Sweden. *Br J Cancer* **70**: 743–748

*© Cancer Research Campaign 1997*